# Complex network analysis of bilateral international investment under de-globalization: Structural properties and evolution

**DOI:** 10.1371/journal.pone.0216130

**Published:** 2019-04-29

**Authors:** Xinxin Xu, Sheng Ma, Ziqiang Zeng

**Affiliations:** 1 Business School, Chengdu University, Chengdu, Sichuan, PR China; 2 Foster School of Business, University of Washington, Seattle, Washington, United States of America; 3 Business School, Sichuan University, Chengdu, Sichuan, PR China; Harbin Institute of Technology, CHINA

## Abstract

As many countries are now seeking to protect their own markets rather than indulge in global trade, this paper examines whether this type of de-globalization behavior has been having any effect on international investment relationships through a systematic analysis of international investment network (IIN) in 127 economies from 2005 to 2016. Unlike previous studies that only analyzed portfolio investment data, the bilateral international investment data were estimated using a matrix-based iteration approach, and the IIN established using complex network theory. Using bilateral international investment data made the results more reliable and somewhat closer to reality. To analyze the structural properties and evolution of the IIN, complex network indicators including a new one named node similarity were developed. The node similarity is defined as the proportion of common relationships of the current economy between two successive years which is useful to reveal the dynamics of the IIN. This paper finds that there are heterogenous and hierarchal properties in the IIN, several economies had a wide range of international investment partners, while most others had only a small range of investment partners and were more likely to form tight groups within the network. The economies in the IIN were tending towards smaller but closer communities, a new trend of regional financial cooperation was developing. The IIN is divided into more communities over time while the top active and central economies often locate in different communities. These findings imply that the structure of the IIN is changing geographically during the de-globalization rather than independent with regions. The regional cooperation has made positive effect on the international investment. The governments should ensure that they continue to support liberal financial policies and to promote better regional financial cooperation.

## Introduction

After the 2008 global financial crisis, the international investment market fluctuated dramatically as many countries sought to disengage their economies from global financial influences [1], which consequently changed the international development landscape. In an attempt to understand these changes, this paper identifies the structural properties of the current international investment network to assess whether these de-globalization trends have been reflected in investment behavior.

Bilateral investment relationships and structural properties can be explicitly described using complex network, which is a successful methodology that has been used to describe the inherent complexities in all areas of science [2], from biology [3–4], transportation [5–6], to economics and finance [7–13].

Therefore, the global economic world can be viewed as complex network made up of many nodes and edges, with each node representing an economy (i.e., country or region), and with each edge between two nodes indicating the presence of economic activities between the two economies. Previous studies have analyzed the world trade web using network modeling frameworks [14–16] in which the world trade network was treated as a binary-network and it was assumed that there was an edge between any two economies if the trade volume was larger than a given threshold. However, as binary networks were unavailable to reflect trade-relationship heterogeneity, weighted world trade network method was developed in each edge was weighted with a trade volume index [14–15]. Serrano and Boguñá first constructed a world trade web and found that it had the typical properties associated with complex networks [16]. The interplay between the topological properties of the world trade web and individual country GDP was subsequently studied, from which it was found that the dynamics of the GDP values were tightly coupled with the world trade web evolution [17].

There have also been many studies on the dynamics associated with cross-border financial investment networks. Song et al. constructed a world investment network using coordinated portfolio investment survey data from 2001 to 2006 and found that there was an increasing globalization trend during this period [18]. Joseph et al. analyzed the cross-border equity and long-term debt securities portfolio investment networks from 2002 to 2012 to measure the robustness of the global financial system and the interdependence of financial markets [19]. Zhang et al. constructed a two-layer international economy network to investigate the world economy from 2001 to 2010 and found that the international investment network community structures were changing dramatically each year [20]. However, most IIN studies have tended to use portfolio investment data which is only one international investment component, primarily because bilateral international investment position data were unavailable. This paper solves the lack of bilateral international investment position data by using the RAS algorithm proposed by the Nobel Prize laureate Richard Stone [21] to reveal the structural properties of the IIN and provide managerial and strategic insights for policy makers.

The remainder of this paper is organized as follows. First, we estimate the bilateral international investment data based on RAS algorithm and then establish the IIN. Then, we construct several indicators to analyze the network structural properties and track the network evolution. Finally, based on the analysis results, we obtain some important insights and conclude with some discussions.

## Establishment of the international investment network

To establish the IIN, international investment position data on 127 economies (see S1 Appendix for a detailed list) were collected after which the bilateral positions between the different economies were estimated using the RAS algorithm as detailed in the following.

### Data collection

The international investment position data for 127 economies from 2005 to 2016 were collected from the Balance of Payment and International Investment Position Statistics (BOP/IIP) provided in the International Monetary Fund (IMF) database, which has data on the total assets and liabilities for each economy in units of millions of US dollars [22]. Economies for which it was not possible to report or calculate the international investment position data from the underlying observations and economies with incomplete data for the observation period were deleted. Therefore, the international investment position data for 127 countries were finally collected. The data collection process complied with all the terms of service for the IMF database. Assets included portfolio investments, direct investments, financial derivations, employee stock options, and other investment and reserve assets, and the liabilities included the same items as for the assets except for reserve assets.

### Bilateral investment estimation based on the RAS algorithm

As most central banks only publish portfolio investment position data, information on bilateral international investment positions is generally unavailable. Therefore, to address this problem, the RAS algorithm was employed to estimate the bilateral international investment positions between the different economies.

**Fig 1:**
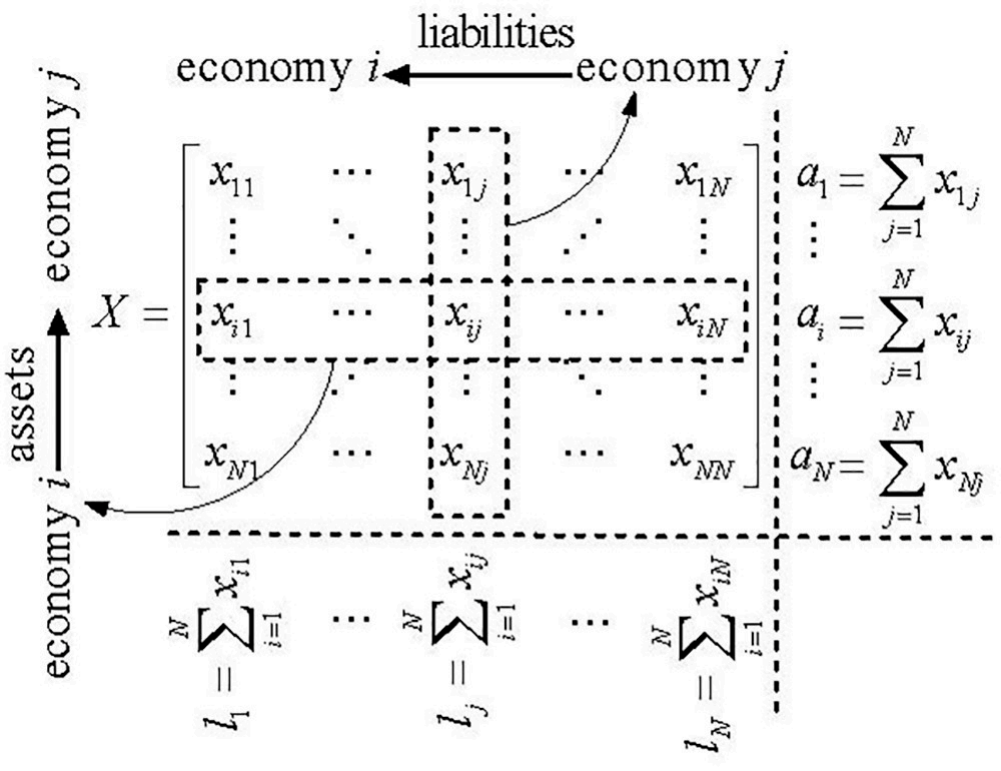
Matrix of assets and liabilities. (a) *x*_*ij*_ are the assets of economy *i* with claims on economy *j*, and *x*_*ij*_ are the liabilities of economy *i* to economy *j*. (b) $a_{i}=\sum_{j=1}^{N}x_{ij}$ is economy *i*’s total assets in the other economies in the international investment markets, and $l_{j}=\sum_{i=1}^{N}x_{ij}$ is *j*’s total liabilities to the other economies in the international investment markets. (c) The total assets *a*_*i*_ and the total liabilities *l*_*j*_ in each economy in each year is known, while the bilateral international investment position *x*_*ij*_ is unobserved.

Suppose that there are *N* economies that may have mutual cross-border investments. The investment relationships in the international investment markets can be represented in an N*N matrix *X* (Fig 1), whereby each element in the matrix *X* indicates that the bilateral international investment position is unknown and needs to be estimated.

Many different non-survey methods have been proposed to produce new estimates such as the entropy method, the location quotient (LQ) method, the EUKLEMS method and the well-known RAS method. Of the many non-survey methods available, the RAS method has proved to be the most popular one because it requires less information and has a reasonable performance. Brand compared the RAS method and Flegg’s location quotient (FLQ) method and found that the RAS method performed better [23]. Even in empirical applications of eight methods on Dutch and Spanish SUTs projections, the RAS method was also found to have superior performance [24]. The main advantages of the RAS method are that it is easy to solve through simple iterations rather than optimization method, and solutions always exist and are unique [25]. Consequently, there have been several modifications to the RAS method, such as the three-stage RAS [26], the two-stage RAS algorithm [27] and the GRAS method proposed by Junius and Oosterhaven [28] to estimate a matrix that contained both positive and negative entries. Not only has the RAS technique been refined but associated applications have emerged in the economic and management fields [29–31].

The RAS method is a matrix balancing method that uses a biproportional technique to estimate a new matrix from an initial matrix by scaling its column and row. The estimation methodology is based on the entropy maximization process introduced by Sheldon and Maurer [32]. The entropy maximization problem is described as follows, “Given a matrix *C*, determine a matrix *X* that is close to matrix *C* and satisfy a given set of linear conditions on its entries” [33]. Where *C* is the real assets and liabilities matrix, and *X* is the estimated assets and liabilities matrix; therefore, it is necessary to minimize the difference between matrix *C* and *X* With appropriate standardization, the total assets *a*_*i*_ and the total liabilities *l*_*j*_ of each economy can be viewed as realizations of the marginal distributions *f*(*a*_*i*_) and *f*(*l*_*j*_), and the bilateral international investment position *x*_*ij*_ as realizations of their joint distribution *f*(*a*_*i*,_ *l*_*j*_); if *f*(*a*_*i*_) and *f*(*l*_*j*_) are independent, then *x*_*ij*_ = *a*_*i*_*l*_*j*_. The independence assumption is based on Sheldon and Maurer [33], which states that entropy maximization subject to the linear restriction implies a bank’s loans and borrowings have stochastic independence, which means that the relative distribution of a bank’s loans across banks has no bearing on the relative intensity with which that bank borrows funds from other banks. The independence assumption is also suitable for countries’ international assets and liabilities. While the independence assumption is not good enough to describe the reality, nevertheless, it deviates as little as possible from the information in our data.

However, the resulting matrix *X* has a non-zero main diagonal that indicates the economies invest with themselves. To resolve this problem, the independence assumption needs to be modified by setting *x*_*ij*_ = 0 for *i* = *j*, which means the relative entropy of matrix *X* with respect to matrix *C* needs to be minimized with elements *x*_*ij*_ = *a*_*i*_*l*_*j*_ for *i* ≠ *j* and *x*_*ij*_ = 0 for *i* = *j*. The problem is mathematically represented as follows:

$$\begin{array}{l} \underset{x_{ij}}{Min}\sum_{i=1}^{N}\sum_{j=1}^{N}x_{ij}\text{ln}\frac{x_{ij}}{c_{ij}}\\ s.t.\left\{ \begin{array}{l} \sum_{j=1}^{N}x_{ij}=a_{i},i=1,\ldots ,N\\ \sum_{i=1}^{N}x_{ij}=l_{j},j=1,\ldots ,N\\ x_{ij}\geq 0 \end{array}\right. \end{array}$$ (1)

where *c*_*ij*_ is an element of matrix *C*, and *x*_*ij*_ is an element of matrix *X*. As *x*_*ij*_ represents the assets and liabilities of an economy, it is non-negative. As the objective function is strictly concave, Model (1) yields a unique solution for the estimated assets and liabilities matrix *X*. When there is a known sum of rows and sum of columns, the RAS method is able to generate a new N * N matrix by pre and post multiplying the initial matrix with the diagonal matrices *R* and *S*. After the adjustment of the parameters *R* and *S*, a new balanced matrix is then created from the original unbalanced matrix. To solve the minimization problem in Model (1), some researchers already proposed special methods, such as Newton-Raphson algorithm [34], geometric programing [35], stochastic optimization [36] and simulated annealing [37]. In this paper the generalization RAS method which has been proved to be a procedure for entropy optimizing by Gorman [38] is employed to solve Model (1). Let $x_{ij}^{\left(0\right)}=c_{ij}$, based on the Karush-Kuhn-Tucker condition [39], it can be derived that:

$$\sum_{j=1}^{N}x_{ij}^{\left(t\right)}e^{\delta }=a_{i},i=1,\ldots ,N,\;\text{and}\;\sum_{i=1}^{N}x_{ij}^{\left(t+1\right)}e^{\delta }=l_{j},j=1,\ldots ,N,$$ (2)

$$x_{ij}^{\left(t+1\right)}=x_{ij}^{\left(t\right)}e^{\delta }$$ (3)

where *δ* ≥ 0. The detailed derivation and proof process can be found in [40]. According to Eqs (2) and (3), substitution of *e*^*δ*^ in Eq (3), then the adjustment of the parameters is implemented based on the following iterative equations:

$$x_{ij}^{\left(t+1\right)}=\frac{a_{i}^{}x_{ij}^{\left(t\right)}}{\sum_{j}x_{ij}^{\left(t\right)}}t=0,2,4,\ldots ,\;\text{and}\;x_{ij}^{\left(t+2\right)}=\frac{l_{j}^{}x_{ij}^{\left(t+1\right)}}{\sum_{i}x_{ij}^{\left(t+1\right)}},t=0,2,4,\ldots ,$$ (4)

where the iterative index *t* (*t* = 0,2,4, ⋯) indicates different steps, respectively. The above steps will iterate until convergence. These steps can be generalized as follows:

$$x_{ij}^{\left(t+1\right)}=x_{ij}^{\left(t\right)}\text{+(}a_{i}^{}-\sum_{j}x_{ij}^{\left(t\right)})\frac{x_{ij}^{\left(t\right)}}{\sum_{j}x_{ij}^{\left(t\right)}},t=0,2,4,\ldots ,$$ (5)

and

$$x_{ij}^{\left(t+2\right)}=x_{ij}^{\left(t\text{+}1\right)}\text{+(}l_{i}^{}-\sum_{i}x_{ij}^{\left(t+1\right)})\frac{x_{ij}^{\left(t+1\right)}}{\sum_{i}x_{ij}^{\left(t+1\right)}},t=0,2,4,\ldots ,$$ (6)

Blien and Graef [40] proved that the above iterations were able to converge to the real assets and liabilities matrix *C* and satisfy the constraints in Model (1) when using the marginals *a*_*i*_ and *l*_*j*_ as the iterative coefficients to adjust the estimated assets and liabilities matrix *X* as shown in Eqs (5) and (6). They also found that the initialization of *X* had significant impacts on the rate of convergence. However, a “good” initial matrix of *X* may result in a significantly reduced iteration number; Therefore, to obtain a “good” initial matrix *X*^(0)^, the following normal distribution with a mean value $\frac{\sum_{i=1}^{N}a_{i}}{N\times N}$ and standard deviation 1 is adopted:

$$\left\{ \begin{array}{l} x_{ij}^{\left(0\right)}=randn*1+\frac{\sum_{i=1}^{N}ai}{N\times N},i\neq j\\ x_{ij}^{\left(0\right)}=0,i=j \end{array}\right.$$ (7)

where $x_{ij}^{\left(0\right)}$ is an element of the initial matrix *X*^(0)^, and *randn* is a normal distributed random number with a mean value of 0 and a standard deviation of 1. As no economy has bilateral investment with itself, for all *i* = *j*, $x_{ij}^{\left(0\right)}$ is set to zero. According to Eqs (5) and (6), if $x_{ij}^{\left(0\right)}=0$, it keeps the value until the end of the iteration as this automatically satisfies the diagonal restriction. The iteration stops if the index *diff* < 0.001, where *diff* is defined as the maximal difference between $x_{ij}^{\left(t+1\right)}$ and $x_{ij}^{\left(t\right)}$:

$$diff=\underset{i,j}{\text{max}}|x_{ij}^{\left(t+1\right)}-x_{ij}^{\left(t\right)}|,t=0,1,2,\cdots ;i=1,2,\ldots N;j=1,2,\ldots ,N$$ (8)

**Fig 2:**
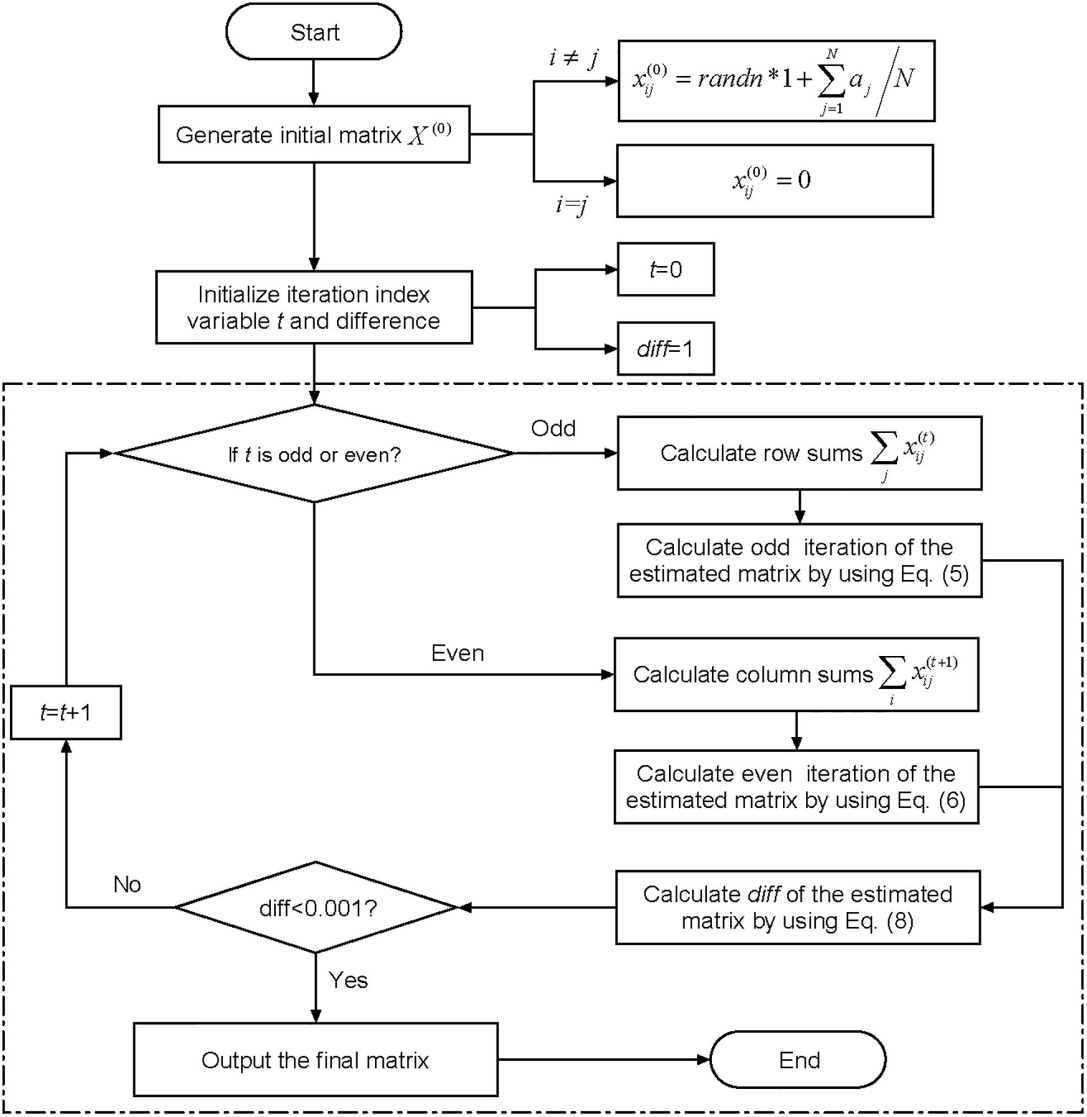
The RAS algorithmic framework.

The RAS algorithm framework is illustrated in Fig 2.

### Layout of the bilateral investment network

**Fig 3:**
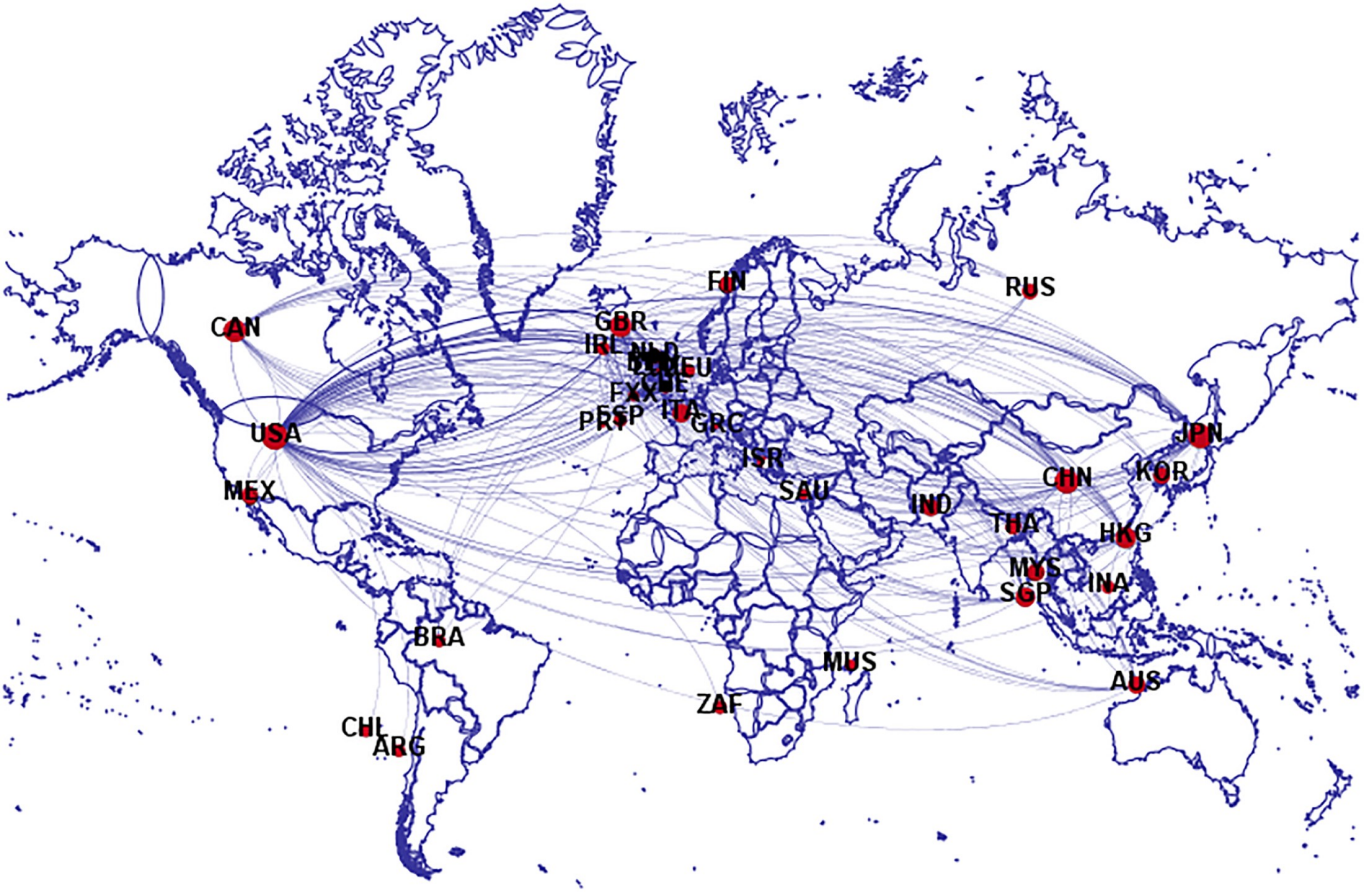
The IIN in which *w*_*ij*_ ≥ 0.02. (a) Each node represents an economy, and the edge between two nodes indicates that there are international investment activities between the two economies. (b) The size of the nodes depends on its degree, and the thickness of the edges depends on its weight. (c) The label for each economy is denoted by the country codes as in the ISO 3166–1 alpha-3 (see S2 Appendix for a detailed list). (d) The definitions for the degree and weight are given in the following section.

The bilateral investment matrixes of 127 economies from 2005 to 2016 were estimated using the RAS algorithm, after which the matrixes were used to build the IIN. To clearly show the bilateral investment network global layout, the method proposed by Cerina et al. [41] was employed to filter the edges with the weight smaller than 0.02, and to label the economies, as shown in Fig 3.

## International investment network analysis

After the IIN was built, several indicators were developed to analyze the structural properties and evolutions of the network from 2005 to 2016.

### Definitions of indicators

The following notations were used to construct the indicators. Let *i* and *j* denote the nodes and *e*_*ij*_ denote an edge that connects economy *i* and economy *j*.

1. The first measure was the degree *d*_*i*_ to determine the number of economies connected to economy *i*:
  $$d_{i}=\sum_{j\in V}e_{ij}$$ (9)
  where *e*_*ij*_ equals 1 if there is an investment relationship between economy *i* and *j* and 0 otherwise and *V* is a set of economies.
2. The investment relationship *e*_*ij*_ was determined from its weight *w*_*ij*_ which was defined using a normalization process to measure the investment strength between economy *i* and economy *j*:
  $$w_{ij}=\frac{x_{ij}-Min_{x_{ij}}}{Max_{x_{ij}}-Min_{x_{ij}}}$$ (10)
  where ${Min}_{x_{ij}}$ and ${Max}_{x_{ij}}$ were the minimum and maximum value of the assets or liabilities.
3. To quantify the importance of an economy in the network, a betweenness index was introduced. Betweenness is a measure of centrality in a graph based on the shortest path and was originally developed by Freeman [42] as a measure to quantify the controlling power of a person in a social network. Following Freeman, the closest relationship was substituted for the “shortest path” concept to measure the extent relationship with the other pairs of economies. Let *σ*_*ij*_ denote the number of closest relationships between *i* and *j*, and let *σ*_*ij*_(*v*) denote the number of closest relationships that pass through node *v* between *i* and *j* (*i* ∈ *V*, *j* ∈ *V*, *v* ∈ *V*). The standard measure of betweenness *b*_*v*_ is as follows:
  $$b_{v}=\sum_{v\neq i\neq j}\frac{\sigma_{ij}(v)}{\sigma_{ij}}$$ (11)
  The betweenness *b*_*v*_ was seen as reflecting the status of each economy in the IIN; that is, a high betweenness value indicated that the economy was located in the center of the network and had important influence in the international investment market.
4. A clustering coefficient was then constructed to describe how the economy’s partners were linked with other economies. The clustering coefficient for economy *i* quantified how close its partners were as a group. The neighborhood *N*_*i*_ for an economy *i* was therefore defined as follows:
  $$N_{i}=\left\{j:e_{ij}\in E\cap e_{je}\in E\right\}$$ (12)
  where *E* was a set of investment relationships. In a directed network, the clustering coefficient *CC*_*i*_ for an economy *i* is the proportion of connections between the economies within its neighborhood divided by the number of connections that probably exist between them; that is:
  $$CC_{i}=\frac{\left|\left\{e_{jk}:j,k\in N_{i},e_{jk}\in E\right\}\right|}{k_{i}(k_{i}-1)}$$ (13)
  where *k*_*i*_ is the number of neighbors to economy *i*, and *K*_*i*_(*K*—1) is the number of connections that exist within neighborhood *N*_*i*_.
5. To analyze the dynamics of the IIN during the whole period, node similarity is proposed which can measure the structural change of the current network compared with a previous year. It defines as the proportion of common relationships of the current economy between two successive years [43]. The node similarity *S*_*i*_(*t*) can be calculated as:
  $$S_{i}(t)=\frac{\left|E(i,t)\cap E(i,t-1)\right|}{\left|E(i,t)\cup E(i,t-1)\right|}$$ (14)
  Where *E*(*i*, *t*) is the set of investment relationships linked with economy *i* at time *t*, and |·|denotes the magnitude of the set.

### Structural properties analysis

**Fig 4:**
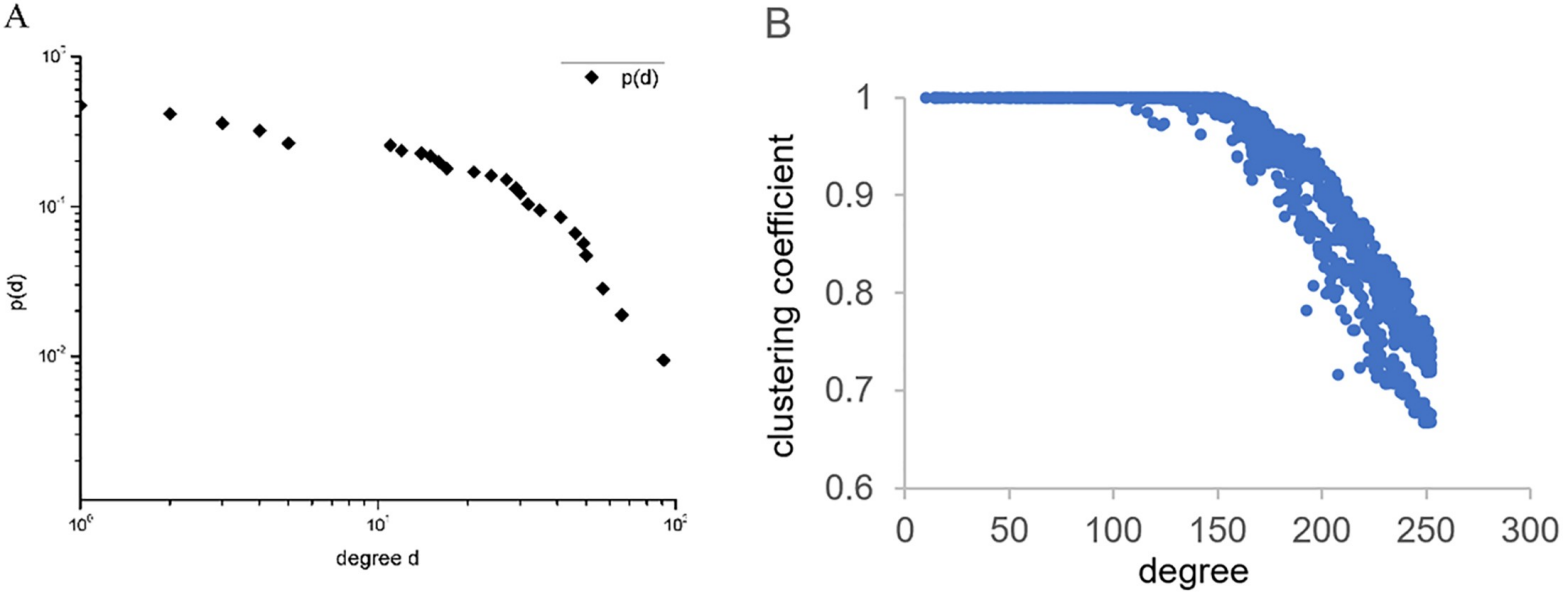
Structural properties of degree and clustering coefficient. (a) Panel A shows the bilogarithmic graph for the complementary cumulative distribution *p*(*d*) and degree *d*_*i*_. (b) Panel B shows the relationship between the degree and the clustering coefficient.

In this section, the statistical properties of the network are given. To analyze the structural properties of degree *d*_*i*_, the complementary cumulative distribution *p*(*d*) was calculated and a bilogarithmic graph of *p*(*d*) and *di* is plotted. Panel A in Fig 4 shows that the distribution of degree *d*_*i*_ had a proportional decreasing trend and a heavy tail, which characterized the power-law. The power-law tail indicated that there was great heterogeneity in the IIN; as can be seen, most economies had a small degree value, while several economies had relatively high degree values.

The relationship between the degree and clustering coefficient data for the 127 economies from 2005 to 2016 are shown on panel B in Fig 4, from which it can be seen that there was a hierarchical structural property in the relationship. The economies with a small degree less than 150 were relative to the highest clustering coefficient, which means that the economies less than 150 investment relationships were more likely to form tight groups in the investment network. However, once the inflection point was exceeded, the clustering coefficient decreased as the degree increased, indicating that the economies with more investment partners were unlikely to form close relationships in the investment network.

### Structural evolution analysis

In this section, the established indicators are applied to analyze the structural evolution of the IIN from 2005 to 2016.

**Fig 5:**
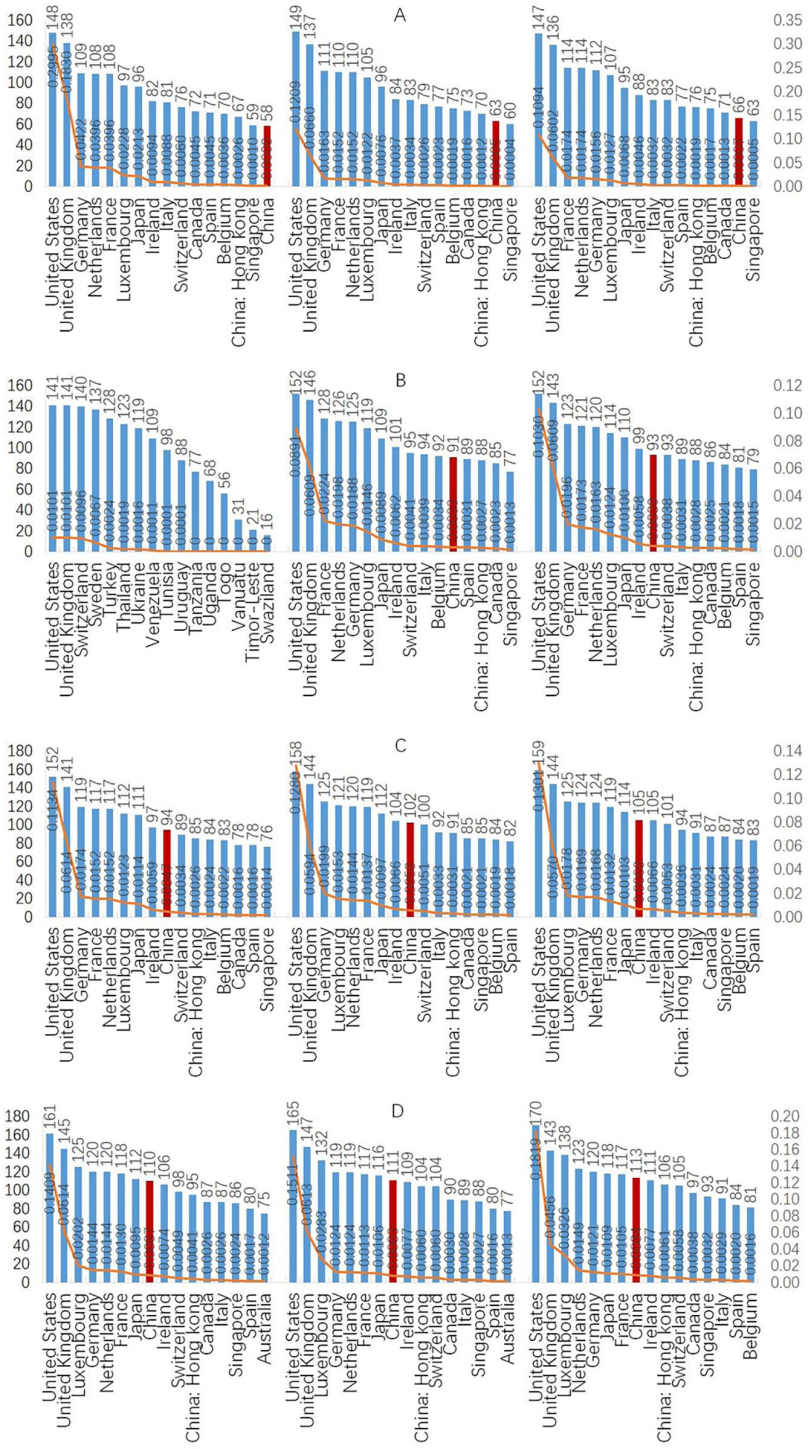
Degree and betweenness evolutions for the top economies from 2005 to 2016. (a) The degree is shown by the histogram and betweenness is shown by the line. (b) In panel A, from left to right shows the degree and betweenness evolutions of the top economies from 2005 to 2007, panel B shows the degree and betweenness evolutions of the top economies from 2008 to 2010, and panel C shows the degree and betweenness evolutions of the top economies from 2011 to 2013, and panel D shows the degree and betweenness evolutions of the top economies from 2014 to 2016. (c) The red histogram represents China. (d) The value of the left coordinate axis is the degree, and the value on the right coordinate axis is the betweenness.

The degree and betweenness evolutions for the top economies from 2005 to 2016 are shown in Fig 5 (Panel A, B, C and D).

**Fig 6:**
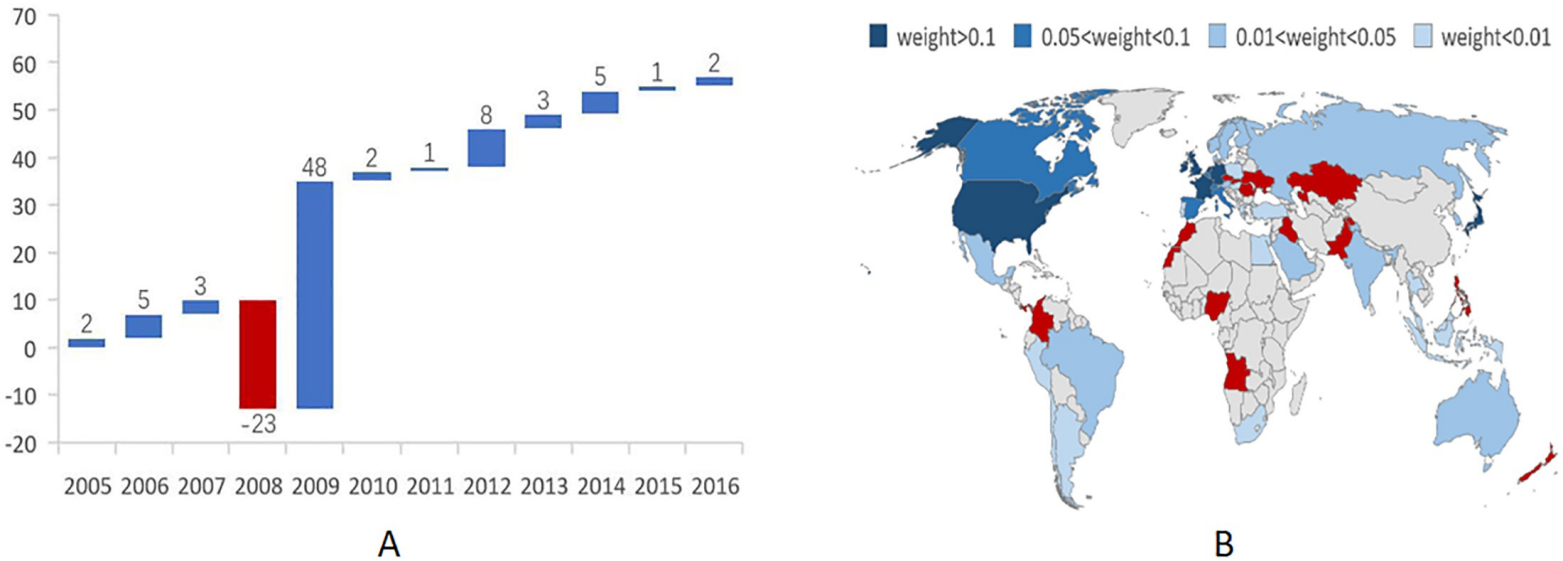
China’s degree change. (a) Panel A shows China’s degree change from 2005 to 2016; the blue histogram indicates an increase, and the red histogram indicates a decrease, and the value above the histogram is the degree change value. (b) Panel B shows China’s international investment partners in 2016, in which the weight value is the sum of the inward investment weight and the outward investment weight for China, and the red plot shows the Belt and Road countries that began international investment activities with China from 2012.

The indicator degree *d*_*i*_ quantifies the activity of an economy in the IIN using the local structural property. As shown in Fig 5, the top active economies quantified by the degree were relatively stable from 2005 to 2016 at the United States, the United Kingdom, Luxembourg, Germany, the Netherlands, France, Japan, China, Ireland, China: Hong Kong, Switzerland, Canada, Italy, Singapore, Spain, and Australia, with the United States and the United Kingdom respectively occupying the top two positions during the whole period. There were some changes, however, in the top active economies; for example, China’s degree increased from 58 in 2005 to 113 in 2016, moving from 16^th^ to 8^th^ in the IIN except for 2008. After 2008, China’s degree increased sharply, which was even more evident from 2012 to 2016 (See Panel A in Fig 6) when it gained 19 new international investment partners, 13 of which were One Belt and One Road (OBOR) countries; New Zealand, the Philippines, Ukraine, Nigeria, Panama, Pakistan, Morocco, Romania, Kuwait, the Slovak Republic, Malta, Egypt, and Iraq (see Panel B in Fig 6), which clearly indicates that the OBOR policy significantly promoted international investment activities with the OBOR countries.

The indicator betweenness *d*_*v*_ reflects the centrality and importance of the economies in the IIN. As shown in Fig 5 by the yellow line, the betweenness ranking had the same trend as the degree, and the structural property remained stable over the whole period, indicating that there was significant heterogeneity in the top important economies. For example, the United States and the United Kingdom had significantly high betweenness over the whole period, while the other economies had maintained relatively lower value.

To further analyze the inter-connectedness in the IIN, the weighted network community structures were visualized using the community detection algorithm developed by Blondel et al. [44]. Which is able to find high modularity partitions in large networks in a short time and reveal the complete hierarchical network community structure.

The algorithm contains two stages that are iteratively repeated to maximize the modularity. In the first stage, each node is assigned to join the community to gain the maximum modularity ΔQ, and the process is repeatedly and sequentially performed for all nodes until no further improvements are achieved. In the second stage of the algorithm, a new network in which the nodes are the communities found in the first stage will be developed. The iteration of the two stages continues until the network is stable. Finally, the maximum modularity is achieved [45,46].

**Fig 7:**
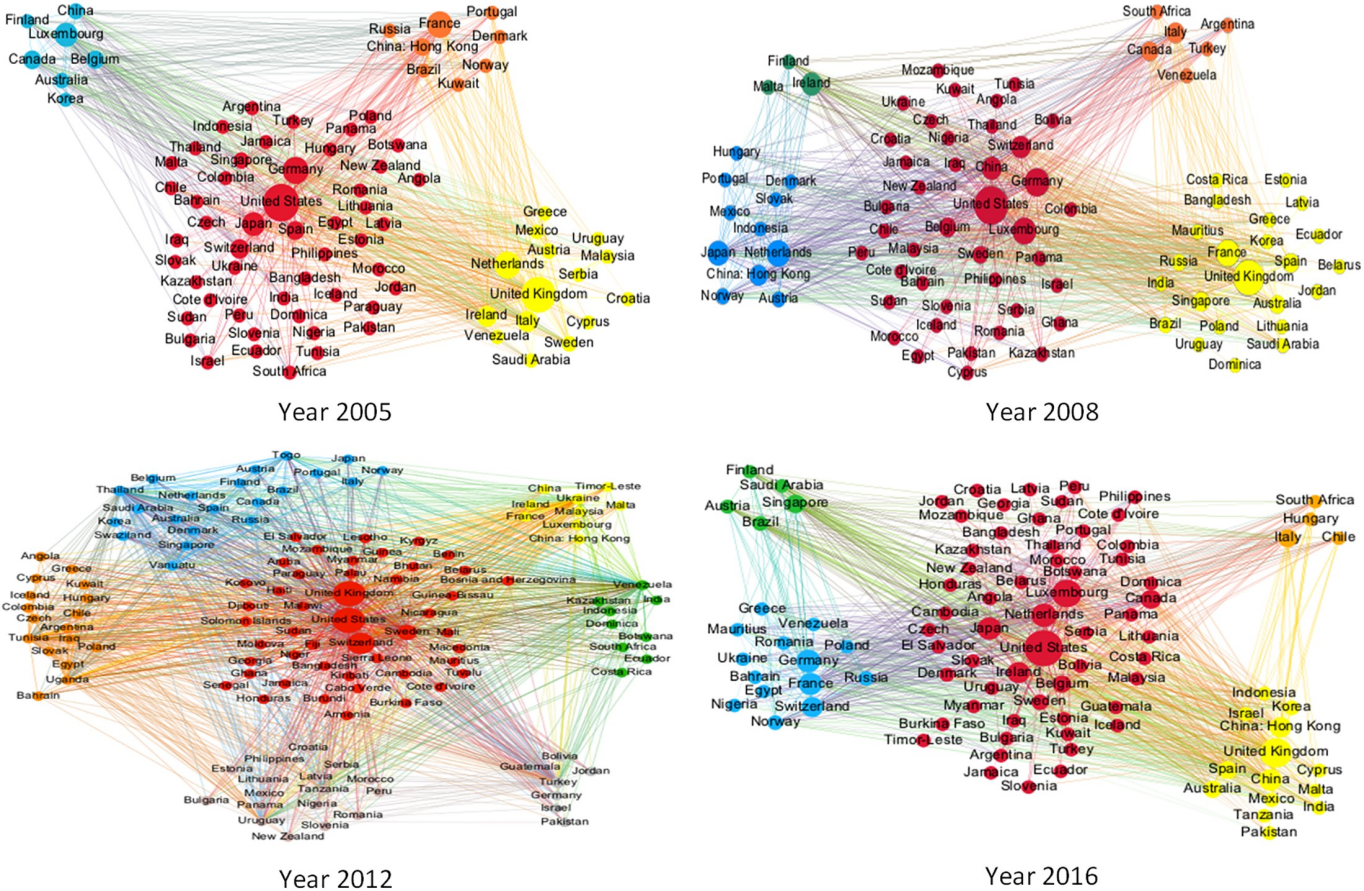
IIN Community structures in 2005, 2008, 2012 and 2016. (a) Each community is represented by a different color; the size of the nodes indicates betweenness. (b) The networks are filtered with edge weights smaller than 0.001.

Based on the weighted IIN in Fig 3, the above community detection algorithm was applied to discover the evolution of the community structures. The first analysis was to determine if there were any significant community structural changes in the IIN before and after the international financial crisis in 2008. It was found that the IIN community structures from 2005 to 2007 were relatively stable, from 2009 to 2011, there were polytropic patterns, and from 2012 onwards, a new stable community structures developed. Therefore, the first and last year (i.e. 2005 and 2016), 2008 and 2012 were selected as the four observation point to more clearly display the dynamic evolution of the IIN community structures. Fig 7 shows the IIN community evolution in 2005, 2008, 2012 and 2016.

From 2005 to 2012 to 2016, the community structures became increasingly complicated with an increasing number of nodes and edges. There was a common property in the community structures in these three years, and the top most active and central economies were located within different groups, which was consistent with the power law of degree and the clustering coefficient that economies with a high degree value are less likely to form groups. The community structure for 2008, however, had the largest number of communities and nodes, and there were 127 economies with edge weights exceeding 0.001, which indicated that the international investment activities in 2008 were extremely active and large.

**Fig 8:**
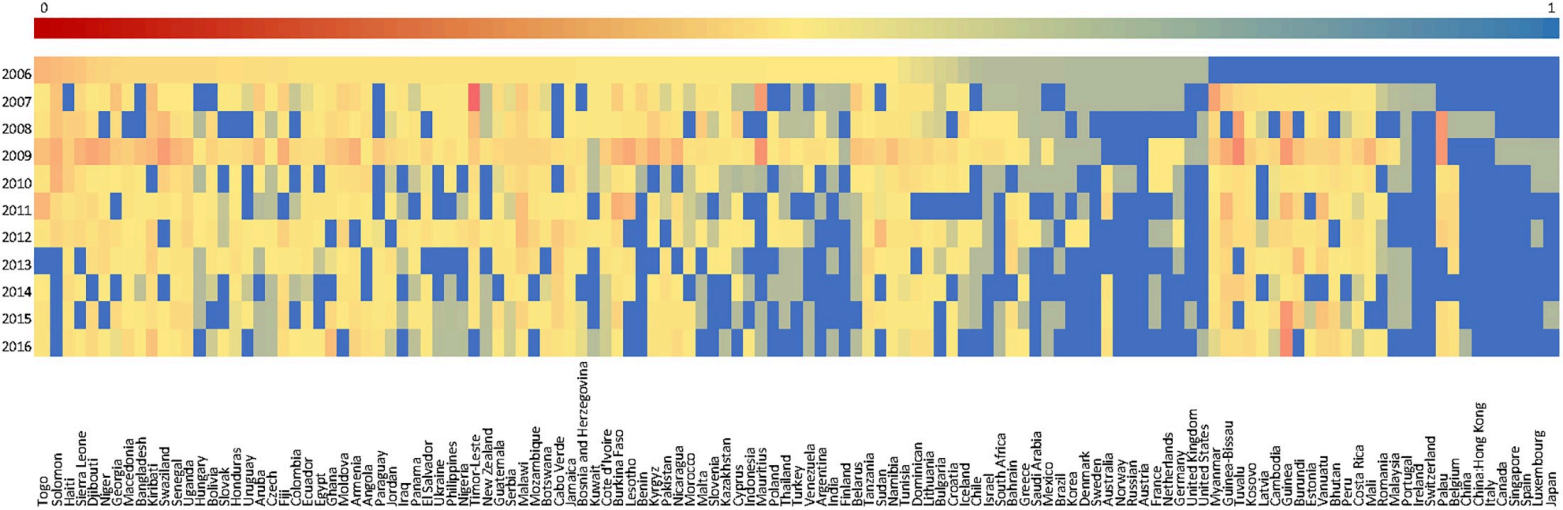
Dynamics of similarity values for all economies. Each pixel represents a similarity value of an economy between two consecutive years, with red color representing low value and blue representing high value.

By applying the node similarity measurement proposed in the previous section, the structural change of the IIN from 2005 to 2016 can be observed in Fig 8. Each pixel represents a similarity value of an economy between two consecutive years, with red color representing low value and blue representing high value. A low value implies a big structural change, and a high value implies a small one. The Fig 8 shows that around 49% of the economies have an average similarity value less than 0.5, which indicates that almost half of the 127 economies encounter at least 50% of structural change. Several economies, such as Switzerland, China, China: Hongkong, Italy, Canada, Singapore, Spain, Luxembourg, and Japan, have maintained a high similarity value during the whole period, which indicates that these economies have considerably stable investment relationships from 2006 to 2016. For the other economies, like Myanmar, Guinea-Bissau, Tuvalu, Kosovo, Latvia, Cambodia, Guinea, Burundi, Estonia, Vanuatu, Bhutan, Peru, and Costa Rica, encountered large investment fluctuations especially in 2009. Compared with the degree analysis results, it can be found that the top active economies mainly have stable investment relationships except the United States, the United Kingdom, Germany, Netherlands, and France. Besides, the evolution of structural similarity of economies implies a hidden regularity. The United States, the United Kingdom, Germany, Netherlands, and France all show high standard deviations with a relatively low average value compared with the other top active economies which have low standard deviations with a relatively high average value.

## Conclusions and discussion

In this paper, an IIN for 127 economies was established using bilateral investment position data estimated using the RAS algorithm, after which the structural properties and evolution of the IIN from 2005 to 2016 were systematically analyzed using statistical indicators.

Compared with studies that have only used the portfolio investment data, the bilateral international investment data allowed for a more detailed investigation of international investment activities. Results for the countries ranking in the IIN were somewhat different from previous research; for example, when portfolio investment data were interrogated, of the top 10 countries and regions from 2001 to 2010, Spain tops the rank from 2003 to 2007, and some larger economies such as China and Russia were not even in the top eight. However, the analysis of the bilateral international investment data revealed that the United States and the United Kingdom occupied the top two position from 2005 to 2016, and China was beginning to playing an increasingly more important role moving from 16th to 8th except for 2008. Therefore, using bilateral international investment data made the results more reliable and somewhat closer to reality.

By analyzing the structural properties and evolution of IIN, some insights can be obtained as follows. Firstly, the strength of financial globalization was decreasing, and a new trend of regional financial cooperation was developing. The degree and clustering coefficient analysis implied that the economies with more investment partners are unlikely to form tight relationships due to the diversified investment, and vice versa. The degree and betweenness analysis indicate that, from 2005 to 2016, the top active economies in the IIN are relatively constant, and the central economies in the IIN are always the United States and United Kingdom. The economies in the IIN were tending towards smaller but closer communities. Furthermore, the regional cooperation has made positive effect on the international investment. Such as the OBOR policy significantly promoted international investment activities within the OBOR countries. While China had made a great change during the period, especially since the OBOR Initiative been proposed, 13 OBOR countries became China’s new international investment partners. Compared with the degree analysis results, the node similarity implies that the top active economies mainly have stable investment relationships except the United States, the United Kingdom, Germany, Netherlands, and France. Finally, by analyzing the community structure of the IIN, this paper also finds that the IIN is divided into more communities over time while the top active and central economies often locate in different communities. These findings imply that the structure of the IIN is changing geographically during the deglobalization rather than independent with regions. Therefore, governments should ensure that they continue to support liberal financial policies and to promote better regional financial cooperation.

## Supporting information

S1 Appendix List of 127 economies (country or region). (DOCX)

S2 Appendix List of economy codes. (DOCX)

S1 File Dataset RAS matrix and weight matrix for 127 economies. (XLSX)
