# Supplementary material for: Complex network analysis of bilateral international investment under de-globalization: Structural properties and evolution
Source: PLoS One. 2019 Apr 29;14(4):e0216130. doi: 10.1371/journal.pone.0216130 (PMC6488084; doi:10.1371/journal.pone.0216130)
Supplement: S1 Appendix — (DOCX) [file pone.0216130.s001.docx]

**S1 Appendix List of 127 economies (country or region)**

| Angola | Costa Rica | India | Moldova | Slovak |
| --- | --- | --- | --- | --- |
| Argentina | Cote d'Ivoire | Indonesia | Morocco | Slovenia |
| Armenia | Croatia | Iraq | Mozambique | Solomon Islands |
| Aruba | Cyprus | Ireland | Myanmar | South Africa |
| Australia | Czech | Israel | Namibia | Spain |
| Austria | Denmark | Italy | Netherlands | Sudan |
| Bahrain | Djibouti | Jamaica | New Zealand | Swaziland |
| Bangladesh | Dominica | Japan | Nicaragua | Sweden |
| Belarus | Ecuador | Jordan | Niger | Switzerland |
| Belgium | Egypt | Kazakhstan | Nigeria | Tanzania |
| Benin | El Salvador | Kiribati | Norway | Thailand |
| Bhutan | Estonia | Korea | Pakistan | Timor-Leste |
| Bolivia | Fiji | Kosovo | Palau | Togo |
| Bosnia and Herzegovina | Finland | Kuwait | Panama | Tunisia |
| Botswana | France | Kyrgyz | Paraguay | Turkey |
| Brazil | Georgia | Latvia | Peru | Tuvalu |
| Bulgaria | Germany | Lesotho | Philippines | Uganda |
| Burkina Faso | Ghana | Lithuania | Poland | Ukraine |
| Burundi | Greece | Luxembourg | Portugal | United Kingdom |
| Cabo Verde | Guatemala | Macedonia | Romania | United States |
| Cambodia | Guinea | Malawi | Russia | Uruguay |
| Canada | Guinea-Bissau | Malaysia | Saudi Arabia | Vanuatu |
| Chile | Haiti | Mali | Senegal | Venezuela |
| China: Hong Kong | Honduras | Malta | Serbia |  |
| China | Hungary | Mauritius | Sierra Leone |  |
| Colombia | Iceland | Mexico | Singapore |  |
