# Supplementary material for: Complex network analysis of bilateral international investment under de-globalization: Structural properties and evolution
Source: PLoS One. 2019 Apr 29;14(4):e0216130. doi: 10.1371/journal.pone.0216130 (PMC6488084; doi:10.1371/journal.pone.0216130)
Supplement: S2 Appendix — (DOCX) [file pone.0216130.s002.docx]

**S2 Appendix List of economy codes**

| Economy code | Economy name | Economy code | Economy name |
| --- | --- | --- | --- |
| USA | United States | FIN | Finland |
| GBR | United Kingdom | IND | India |
| LUX | Luxembourg | SAU | Saudi Arabia |
| NLD | Netherlands | INA | Indonesia |
| DEU | Germany | GRC | Greece |
| FXX | France | POL | Poland |
| JPN | Japan | PRT | Portugal |
| CHN | China, P.R.: Mainland | ZAF | South Africa |
| IRL | Ireland | THA | Thailand |
| CHE | Switzerland | TUR | Turkey |
| HKG | China, P.R.: Hong Kong | CHL | Chile |
| CAN | Canada | HUN | Hungary |
| ITA | Italy | ISR | Israel |
| SGP | Singapore | MYS | Malaysia |
| BEL | Belgium | MUS | Mauritius |
| ESP | Spain | ARG | Argentina |
| AUS | Australia | COL | Colombia |
| SWE | Sweden | CYP | Cyprus |
| NOR | Norway | CZE | Czech Republic |
| BRA | Brazil | MLT | Malta |
| KOR | Korea, Republic of | NZL | New Zealand |
| RUS | Russian Federation | BHR | Bahrain, Kingdom of |
| DNK | Denmark | KAZ | Kazakhstan |
| AUT | Austria | KWT | Kuwait |
| MEX | Mexico | PHL | Philippines |
